# Assessment of Operator Reliability in Ultrasound of the Median and Ulnar Nerve Using Bland-Altman Analysis

**DOI:** 10.3390/diagnostics11112134

**Published:** 2021-11-17

**Authors:** Jörg Philipps, Hannah Mork, Maria Katz, Mark Knaup, Kira Beyer, Kristina Spies, Johannes Corbach, Peter D. Schellinger

**Affiliations:** Department of Neurology and Neurogeriatrics, Ruhr-University Bochum, Johannes-Wesling-Klinikum Minden, UK RUB, Hans- Nolte Str. 1, D-32429 Minden, Germany; hannah.mork@rub.de (H.M.); maria.katz@rub.de (M.K.); mark.knaup@rub.de (M.K.); kira.beyer@rub.de (K.B.); kristina.spies@rub.de (K.S.); johannes.corbach@muehlenkreiskliniken.de (J.C.); peter.schellinger@rub.de (P.D.S.)

**Keywords:** nerve ultrasound, cross-sectional area, reliability, training effect, Bland–Altman agreement analysis

## Abstract

Currently, there is no standardized method to evaluate operator reliability in nerve ultrasound. A short prospective protocol using Bland–Altman analysis was developed to assess the level of agreement between operators with different expertise levels. A control rater without experience in nerve ultrasound, three novices after two months of training, an experienced rater with two years of experience, and a reference rater performed blinded ultrasound examinations of the left median and ulnar nerve in 42 nerve sites in healthy volunteers. The precision of Bland–Altman agreement analysis was tested using the Preiss–Fisher procedure. Intraclass correlation coefficients (ICC), coefficients of variation, and Bland–Altman limits of agreement were calculated. The sample size calculation and Preiss–Fisher procedure showed a sufficient precision of Bland–Altman agreement analysis. Limits of agreement of all trained novices ranged from 2.0 to 2.9 mm^2^ and were within the test’s maximum tolerated difference. Ninety-five percent confidence intervals of limits of agreement revealed a higher precision in the experienced rater’s measurements. Operator reliability in nerve ultrasound of the median and ulnar nerve arm nerves can be evaluated with a short prospective controlled protocol using Bland–Altman statistics, allowing a clear distinction between an untrained rater, trained novices after two months of training, and an experienced rater.

## 1. Introduction

The reliability and validity of nerve ultrasound has been analyzed in healthy individuals [1,2], cadavers [3], and patients [4]. Reliability between different work groups for measurements of the nerve cross-sectional area (CSA) has been assessed in a prospective multicenter study [5]. Garcia-Santibanez et al. [6] analyzed the effect of examiner training in trainees with different levels of experience and found that two months of training are sufficient to reliably measure upper limb nerves and they recommend assessing training results individually for each operator.

Skills in nerve ultrasound consist of finding the correct structure, positioning the transducer in the correct angle, taking a picture with appropriate contrast, and tracing the limits of the nerve with the tracing tool. All these skills need appropriate training and can contribute to higher reliability, both in trainees and in more experienced examiners. With gaining importance of nerve ultrasound in the diagnostic workup of diseases of the peripheral nervous system, the method is performed by a growing number of physicians and other trained personnel, which makes constant reliability within and in between teams an important issue. Currently, a standardized method to evaluate operator performance in nerve ultrasound does not exist.

The aims of the present study were:

- To develop a short standardized protocol to assess the level of agreement between operators based on sample size and precision calculations of Bland–Altman analysis;
- To use this protocol in a team of operators with different expertise levels in nerve ultrasound and compare the results of Bland–Altman agreement analysis to the calculation of intraclass correlation coefficients (ICCs) and coefficients of variation.

## 2. Materials and Methods

### 2.1. Participants

Seven healthy volunteers without clinical signs of neuropathy and normal nerve conduction studies (motor, sensory, and F-wave) of the left median and ulnar nerve and six operators with different expertise levels in nerve ultrasound and durations of training participated in the study.

Raters’ training consisted of at least 20 neuromuscular ultrasound examinations per month, theoretical instructions, and presentations on nerve ultrasound. Bedside hands-on training was performed by an experienced examiner (rater 4) under supervision of an examiner with ten years of experience in nerve ultrasound (rater 5). This rater served as local laboratory reference in the absence of a gold standard. A medical student with experience in general ultrasound and excellent anatomical knowledge but without training in nerve ultrasound served as the control. Raters 1, 2, and 3 were trained novices with two months of experience in nerve ultrasound. Rater 4 was as an experienced rater who has performed nerve ultrasound in adults and children for two years.

### 2.2. Ultrasound Examination

The raters performed nerve ultrasound CSA measurements of the left median and ulnar nerve at predefined localizations with an Affiniti 50 (Philips, Amsterdam, The Netherlands) device using a 5 to 18 MHz linear array transducer. The ulnar nerve was measured at Guyon’s canal, 15 cm proximal to Guyon’s canal, and in the upper arm 10 cm proximal to the elbow. The median nerve was measured at the carpal tunnel, in the forearm 15 cm proximal to the carpal tunnel, and in the upper arm 10 cm proximal to the elbow. Raters used a tape measure, but ink marking on the skin of the volunteer was not permitted.

The transducers position was perpendicular to avoid anisotropic effects and to obtain the correct CSA. For CSA measurements in mm^2^, the nerves were visualized in a transverse plane and measured within the inner border of the hyperechoic epineurium using the free-hand tracing function. The raters performed a blinded second measurement of all nerve sites in all volunteers between 1 and 14 days after the first measurement.

### 2.3. Blinding

Volunteers were placed behind a perforated screen. The left arm was the only part of the body visible to the ultrasound operator. Volunteers were examined in a randomized order, changing between one rater and another.

### 2.4. Statistical Calculations

The means and SDs of the CSA of each nerve site and rater were calculated separately. In order to avoid the influence of repeated measurements of the same nerve site, only the first measurement was used. Spearman’s rho was calculated to assess the influence of the volunteers’ age and body mass index (BMI) on the differences of the CSA measurements between each rater and the reference rater.

The measurements of different unilateral single nerve sites in seven volunteers were considered as sufficiently independent measurements. A sample size estimation (type I error = 0.05, type II error = 0.2) was carried out to find out the minimum number of observations required for Bland–Altman agreement analysis [7]. Based on the results of Garcia-Santibanez [6] and our own previous data [8], an assumption of an expected mean of differences of 0.1, SD of 1, and a maximum tolerated difference of 3 mm^2^ was made.

After pairing the data of a given rater’s first measurement to the reference (rater 5), the SD of the differences was compared to the distribution of the SDs of the differences from 1000 random mispairings of that data (Preiss–Fisher procedure) to find out whether the measurement range was wide enough to produce meaningful results in a Bland–Altman agreement analysis [9].

Before performing the Bland–Altman agreement analysis, the differences of the measurements at all nerve sites were tested for normal distribution using the Shapiro–Wilk test.

To assess operator reliability, Bland–Altman agreement analysis was based on the first measurements of a given rater compared to the first measurements of the reference (rater 5). Bland–Altman plots were produced including the 95% CI of the lower and upper limits of agreement (LoA), the mean difference, and the predefined maximum tolerated difference of 3 mm^2^. The 95% CI of the LoA was calculated approximatively as LoA +/− 1.96*√(3*SD^2^/n).

Exact parametric CIs considered as a pair were calculated using the method and coefficients provided by Carkeet [10].

For the upper LoA, exact 95% confidence intervals were calculated as mean +1.6280*SD and mean +2.5335*SD. For the lower LoA, exact 95% confidence intervals were calculated as mean −1.6280*SD and mean −2.5335*SD.

Interrater ICCs were calculated as two-way random ICCs for absolute agreement based on the first measurement of a given rater and the first measurement of the reference rater.

Intrarater reliabilities were calculated as two-way random ICCs for absolute agreement based on the first and the second measurement of a given rater.

Coefficients of variation were calculated as 100*(SD of differences between a given rater and reference/mean) [within-subject standard deviation method].

STATA version 16 (StataCorp, College Station, TX, USA) and SciStat Version 2.8.46 (MedCalc Software Ltd., Ostend, Belgium) were used for statistical analyses.

## 3. Results

### 3.1. Demographics

The mean age of the 5 female and 2 male volunteers was 32.5 years (range 24–53, SD 6.4); their mean BMI was 23.3 (range 18.8–27.1, SD 3.1).

The means and SDs of the nerve CSA (Rater 1–5) of each nerve site are displayed in Table 1.

#### 3.1.1. Sample Size and Normal Distribution Analysis

The sample size estimation yielded a minimum of 35 observations to perform a Bland–Altman agreement analysis. Differences of measurements between raters showed a normal distribution in the Shapiro–Wilk test. Spearman’s rho showed no significant correlation between age or BMI and the differences of CSA measurements between raters.

#### 3.1.2. Preiss–Fisher Procedure

**Figure 1:**
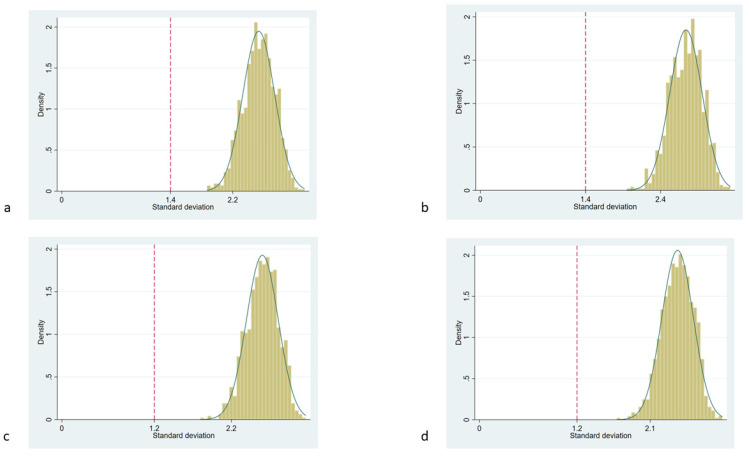
Results of the Preiss–Fisher procedure. The Preiss–Fisher diagrams show the SD of the differences of the Bland–Altman agreement analysis (red dotted line) and the distribution of the SDs of the differences from 1000 random mispairings of the rater/reference data with the 5th percentile value. (**a**) Rater 1/reference, (**b**) rater 2/reference, (**c**) rater 3/reference, and (**d**) rater 4/reference.

The Preiss–Fisher procedure’s 5th percentiles were 2.4, 2.2, 2.4, 2.2, and 2.1 for the control and Raters 1–4 respectively. The observed SD of differences of the Bland–Altman agreement analysis was lower than the Preiss–Fisher procedure’s 5th percentiles in all raters (Preiss–Fisher plots for rater 1–4 see Figure 1).

#### 3.1.3. Bland–Altman Analysis

**Figure 2:**
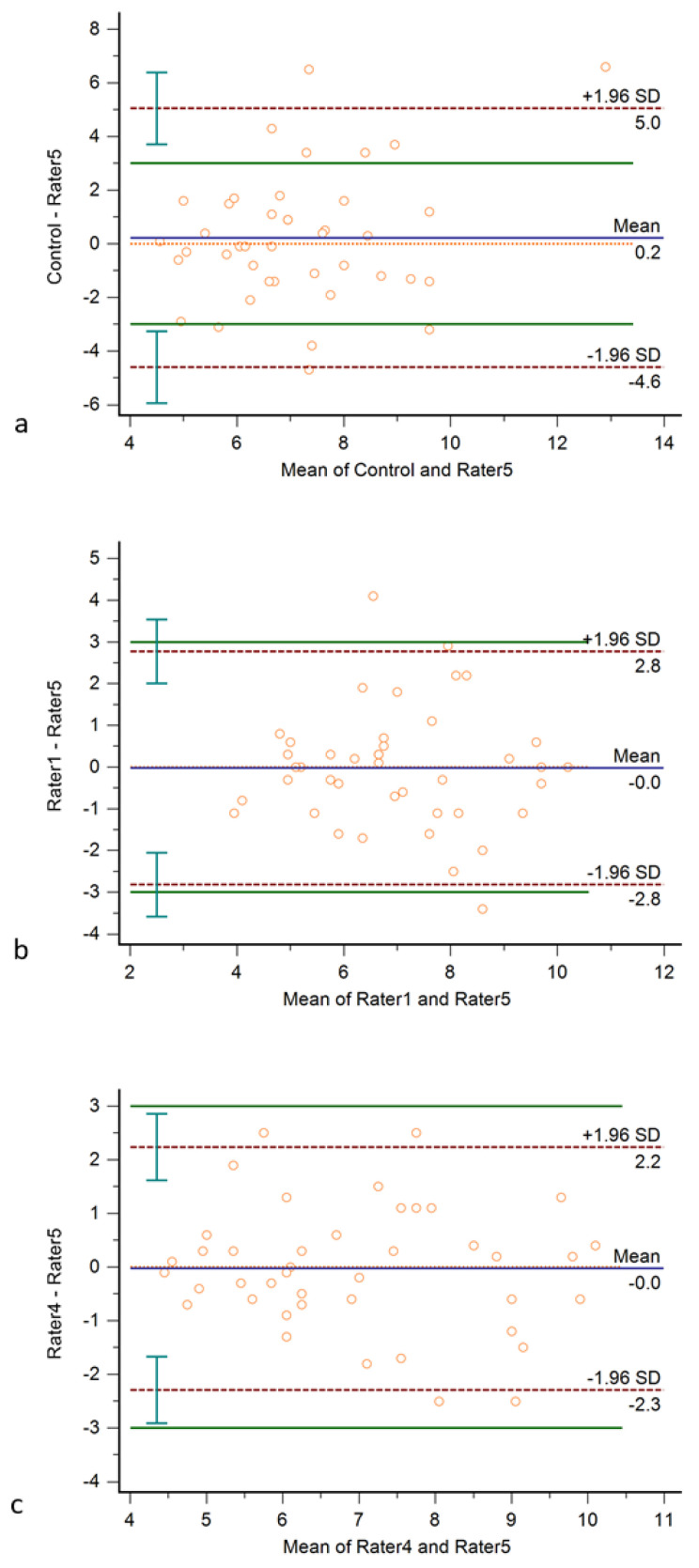
Bland–Altman plots of (**a**) control rater/reference, (**b**) trained novice/reference, and (**c**) experienced rater/reference. Bland–Altman plots include the mean difference (blue), limits of agreement (red dotted line) with an approximate 95% CI of the limits of agreement, and the maximum tolerated difference (green line).

Bland–Altman plots with the approximate CI of the LoA are displayed in Figure 2.

Bland–Altman statistics including approximate and exact parametric CI, two-way random ICCs for inter- and intrarater reliabilities, and coefficients of variation are shown in Table 2. The control rater’s LoA was outside the maximum tolerated difference, the trained novices’ LoAs were inside the maximum tolerated difference, and both the approximate and the exact 95% CI of the LoA of the experienced rater were inside the maximum tolerated difference.

## 4. Discussion

The results of the present study indicate:

- That operator reliability in nerve ultrasound of the median and ulnar nerve can be evaluated with a short prospective controlled protocol using Bland–Altman statistics;
- That a clear distinction between an untrained rater, trained novices after two months of training, and an experienced rater can be made using Bland–Altman agreement analysis.

In order to evaluate operator reliability with a minimum number of volunteers and in a short time, a sample size calculation based on Bland–Altman statistics was performed showing a sufficient number of measurements in the present study. Calculations in the present study are based on different nerve sites’ measurements from each volunteer. This is common practice in studies reporting interrater reliabilities in nerve ultrasound [1,11,12,13]. In the present study, volunteers were selected in a narrow range of BMI and age; no significant influence of age and BMI on measurement differences between raters was detected. The absence of an influence of age and BMI on the level of agreement is in line with the observations of Garcia-Santibanez [6], who found no such influence even in patients. As a high variance between the objects of assessment may have an influence on interrater reliability [14], we selected six nerve sites of the median and ulnar nerve with a known similar SD and high reproducibility [5] between trained operators. The high reproducibility of the selected nerve sites was confirmed in a recent meta-analysis [15]. Blinded measurements of the selected nerve sites can, therefore, be used to assess an operator’s capability to produce reliable results.

Bland–Altman agreement analysis has been extensively studied and is a widely used method to assess agreement between raters [9]. Although a very robust method to estimate agreement between raters, Bland–Altman agreement analysis may produce irrelevant results if the differences between the rated objects or the total number of measurements is low. To our best knowledge, an evaluation of the precision of Bland–Altman agreement analysis in nerve ultrasound has not yet been published. The precision of the Bland–Altman agreement analysis was tested using the Preiss–Fisher procedure, showing a sufficient precision of the test in all raters’ measurements (Figure 1). The maximum tolerated difference of 3 mm^2^ was based upon the differences of up to 2 mm^2^ observed after two months of training in the study of Garcia-Santibanez [6]; in the present study, it should be understood as a very conservative threshold of non-tolerability. The difference of LoA between the control rater and any of the trained operators (Figure 2) shows that this threshold marks a clear difference.

Further steps in a process of establishing a standardized protocol to assess operator performance could include a Delphi survey defining the desired maximum tolerated difference, expected mean of differences, and SD. The possibility of defining and adjusting these limits based on specific clinical considerations is another advantage of the Bland–Altman method. Using the described method to evaluate training effects, we observed that, in Bland–Altman agreement analysis, all trained novices’ LoAs were within the maximum tolerated difference, but only the experienced operator showed an approximate and exact 95% CI of the LoAs within those limits (Figure 2). Recently, the use of tolerance intervals has been proposed as an alternative to the 95% CI of LoA [16]. However, the majority of authors [9,10] recommend the use of LoAs including exact parametric 95% CI as calculated in the present study. The protocol could be extended to other motor [17] or sensory nerves [18] of the upper and lower extremity using the established scanning protocols.

Training intensity in the present study was comparable to the study of Garcia Santibanez [6]. Accordingly, ICCs were high in trained novices and an experienced operator (rater 4, see Table 2) in both studies. Bland–Altman analyses offer a clear distinction between trained novices and an experienced operator.

There are limitations of the present study. The study is limited to the median and ulnar nerve. Neither the applicability of Bland–Altman agreement analysis nor the individual results of operator reliability in volunteers with a low and relatively uniform BMI can be generalized or applied to lower limb nerves. The number of nerve sites analyzed is low, due to the aim of developing a short and easily repeatable method and according to the sample size calculation. A standardized protocol does not replace individual supervision, especially in nerve sites with low inter- and intrarater reliability and in difficult patient settings.

## 5. Conclusions

In a neurological or neurosurgical clinic with high numbers of patients and a team of changing ultrasound operators with different expertise, a standardized protocol to assess the reliability of nerve ultrasound CSA measurements of the median an ulnar nerve can help to maintain a documented level of agreement in daily routine, which is especially important in follow-up examinations. ICCs, coefficients of variation, and Bland–Altman agreement analysis offer a different perspective on agreement. Bland–Altman agreement analysis allows a clear distinction between an untrained rater, trained novices, and experienced raters and can, therefore, facilitate the assessment of operator performance in nerve ultrasound.

## Figures and Tables

**Table 1:** Results of nerve cross-sectional area measurements (mm^2^).

Nerve Site	Rater Mean (SD)	Rater 2 Mean (SD)	Rater 3 Mean (SD)	Rater 4 Mean (SD)	Rater 5 Mean (SD)
Median nerve at the carpal tunnel CSA	8.01 (1.30)	7.49 (1.71)	8.20 (2.23)	8.94 (1.05)	7.90 (1.32)
Median nerve in the forearm CSA	7.40 (1.68)	6.29 (1.22)	6.80 (1.34)	6.33 (1.16)	6.80 (1.27)
Median nerve in the upper arm CSA	7.93 (1.76)	8.77 (1.13)	8.63 (1.78)	8.49 (1.12)	8.86 (1.37)
Ulnar nerve at Guyon’s canal CSA	5.71 (1.46)	4.83 (0.86)	5.06 (1.12)	5.54 (0.79)	5.21 (0.67)
Ulnar nerve in the forearm CSA	5.33 (1.21)	5.34 (1.51)	5.24 (1.49)	5.70 (0.73)	5.41 (0.89)
Ulnar nerve in the upper arm CSA	7.40 (1.29)	7.13 (1.49)	6.74 (1.55)	6.77 (1.38)	7.74 (1.77)

CSA: cross-sectional area; SD: standard deviation.

**Table 2:** Bland–Altman statistics, intraclass correlation coefficients for inter-and intrarater reliabilities, and the coefficients of variation.

Rater	Training Time	Bland–Altman Agreement Analysis Mean Differences	Bland–Altman Agreement Analysis Limits of Agreement (Approximate 95% CI) [Exact 95% CI]	Interrater Reliability Intraclass Correlation Coefficients (95% CI)	Coefficient of Variation	Intrarater Reliability Intraclass Correlation Coefficients (95% CI)
control	none	0.2	−4.6/5.0 (−5.9/6.3) [−6.0/6.4]	0.23 (0–0.49)	26.3%	0.05 (0–0.35)
1	2 months	0.0	−2.8/2.8 (−3.6/3.6) [−3.7/3.7]	0.68 (0.47–0.81)	14.3%	0.73 (0.55–0.85)
2	2 months	−0.3	−2.7/2.0 (−3.4/2.7) [−3.5/2.8]	0.77 (0.60–0.87)	12.9%	0.72 (0.54–0.84)
3	2 months	−0.2	−2.9/2.5 (−3.7/3.2) [−3.7/3.3]	0.75 (0.58–0.86)	13.9%	0.62 (0.40–0.78)
4	2 years	−0.0	−2.3/2.2 (−2.9/2.8) [−2.9/2.9]	0.78 (0.62 to 0.88)	11.6%	0.89 (0.81–0.94)

95% CI: 95% confidence interval. Bland–Altman approximate and exact 95% CI of limits of agreement are indicated as the upper margin of the upper LoA and lower margin of the lower LoA.

## Data Availability

The data presented in this study can be retrieved from osf.io/67dpr Identifier: DOI 10.17605/OSF.IO/67DPR.

## Abbreviations

BMI	body mass index
CSA	cross-sectional area
ICC	intraclass correlation coefficients
LoA	limits of agreement
CI	Confidence interval
